# Comprehensive Study of Different Expressed Genes and Their Functional Modules in Anesthesia for Off-Pump Coronary Artery Bypass Grafting

**DOI:** 10.1155/2020/8062902

**Published:** 2020-07-04

**Authors:** Hui Zhou, Wang Min, Zhihua Zhu

**Affiliations:** ^1^Department of Anesthesia, China-Japan Union Hospital of Jilin University, Changchun, Jilin Province, China; ^2^Department of Pathology, Jilin Provincial Cancer Hospital, Changchun, Jilin Province, China

## Abstract

**Purpose:**

The effect of preoperative anesthesia on coronary artery bypass grafting without extracorporeal circulation is not apparent. We want to investigate the effects and molecular mechanisms of two anesthesia methods on the treatment of coronary artery bypass grafting (OPCABG) under extracorporeal circulation. *Patients and Methods*. The data of inhaled anesthesia and intravenous anesthesia before coronary artery bypass grafting were downloaded from the GEO database, and the differences were analyzed with the control group. The combination of multiple analytical methods can decipher the mechanism of anesthesia on surgery, including protein interaction network analysis, enrichment analysis, and regulatory subprediction.

**Results:**

This study obtained 6699 differential genes under two kinds of anesthesia before OPCABG. By constructing a protein interaction network of differentially expressed genes, we obtained 14 functional module networks. By predicting regulators of functional module genes, we revealed a series of ncRNAs (miR-129-5p, miR-340-5p, and miR-410-3p) and transcription factors (VHL and YBX1).

**Conclusion:**

Based on functional module network analysis, we identified the effects of preoperative inhalation anesthesia and intravenous anesthesia on OPCABG, which provides a valuable theoretical reference for subsequent clinical studies.

## 1. Introduction

Off-pump coronary artery bypass grafting (OPCABG) is the latest surgical procedure in cardiac surgery [1]. OPCABG has proven to be an effective surgical revascularization program [2]. Surgery has determined the safety of OPCABG and its short-term efficacy, but long-term clinical outcomes are uncertain. OPCABG is still the treatment of choice in modern cardiac surgery, considering that surgical practice is continuously changing [3]. Compared with cardiopulmonary bypass grafting, OPCABG has particular advantages in reducing postoperative complications, including systemic inflammatory response, myocardial injury, renal injury, and brain injury [4]. In theory, OPCABG can improve long-term survival by reducing perioperative complications, such as stroke, myocardial damage, cardiac-related mortality, and neurocognitive impairment [5].

Most importantly, OPCABG reduces the risk of neurological complications compared with extracorporeal coronary artery bypass grafting [6]. OPCABG, a high-tech myocardial revascularization program, can reduce sympathetic stress and improve hemodynamic changes [7]. OPCABG has become the standard surgery in Japan [8]. The transplantation of OPCABG in high-risk patients remains controversial, but studies have shown its potential benefits. Therefore, in high-risk patients, the application of its technology is still valued and studied [9]. For patients undergoing OPCABG surgery, the measurement of anesthesia depth is clinically significant and can achieve the effects of avoiding intraoperative perception and cardiac suppression. The recent introduction of entropy as a monitor of anesthesia depth determines the amount of anesthetic used in patients with OPCABG surgery [10]. Given the high hemodynamic instability and the risk of organ damage in OPCABG surgery, xenon anesthesia has become an attractive aesthetic with good hemodynamic and organ protection properties [11]. Studies have shown that during OPCABG, compared with total intravenous anesthesia, volatile anesthetics can reduce myocardial damage, which is measured via cardiac troponin levels [12]. Compared with the sputum, the anesthetic function of sevoflurane in OPCABG reduces the risk of OPCABG surgery due to vasopressin [13]. Changes in magnesium levels can cause abnormal blood coagulation, leading to bleeding complications after OPCABG [14]. In OPCABG, ropivacaine and fentanyl can be used to temporarily reduce arterial pressure, optimize myocardial function, and affect perioperative fluid and vasoactive treatment [15]. Recent studies have shown that dexmedetomidine, as an effective adjuvant, can reduce hospital stays in patients receiving OPCABG [16]. Different expressed genes and their functional modules in anesthesia for OPCABG need to be further explored. Based on a functional modular network, we analyze the effects of preoperative inhalation anesthesia and intravenous anesthesia on OPCABG to explore the underlying molecular mechanisms. It provides not only valuable research directions but also abundant valuable theoretical references for further research on the clinical application of anesthesia.

## 2. Material and Methods

### 2.1. Data Resource

The STRING database [17] is a search tool specifically designed to retrieve protein-protein interactions, which provides comprehensive insight into the currently available PPIs and can, therefore, be used for a wide range of PPI analyses. In this study, the STRING database was utilized for the access of all human protein interaction data, and we set the interaction score > 900, involving 405,916 interaction pairs of 10,514 proteins.

### 2.2. Differential Expression Analysis

We collected an expression microarray data set for inhaled anesthesia and intravenous anesthesia before coronary artery bypass grafting from the NCBI Gene Expression Omnibus database [18], numbered GSE4386. In this data matrix, patients scheduled for off-pump CABG were randomized into a group with the anesthetic gas sevoflurane (*n* = 10) or the intravenous anesthetic propofol (*n* = 10). Atrial samples were collected at the beginning and end of bypass surgery to determine gene expression profiles. A two-difference analysis (anaerobic gas sevoflurane-intravenous anesthesia with propofol) was performed on the collected samples and calculated using “limma” package in R [19]. The 6699 differential genes were combined to construct an expression profile matrix for nonexternal coronary artery bypass grafting.

### 2.3. Functional and Pathway Enrichment Analysis

We explored the function of differential genes and their involved signaling pathways. We performed a GO function (with a cutoff *p* value of 0.01 and a cutoff *q* value of 0.01) and a KEGG pathway enrichment analysis (with a cutoff *p* value of 0.05 and a cutoff *q* value of 0.2) for the 14 modules of the gene using clusterProfiler [20]. We screened the process-related functions and pathways during anesthesia for OPCABG and mapped the bubbles.

### 2.4. Identification of Transcription Factors (TFs) and Modules of ncRNA Regulation

We defined the pivot regulator as a kind of modulator that significantly affects the functional modular network of off-peak coronary artery bypass grafting. Noncoding genes and TFs often drive the transcription, as well as the posttranscriptional regulation of the genes. We downloaded the relevant TF target data from the TRRUST database [21] and finally obtained 33 interaction pairs of 25 TFs. Afterwards, ncRNA-mRNA data was accessed from the RAID database [22], and 660 interaction pairs including 486 ncRNAs were also obtained. Pivot analysis was then performed according to these interactions to further identify the regulatory effects of these TFs and ncRNAs in the module. The saliency of the interactions among the drivers and the modules is calculated based on the hypergeometric test. TF and ncRNA are pivoted for the essential regulatory module, with a *p* value < 0.01 as the screening standard. We performed a statistical analysis of the pivot, and the central function of the more dysfunctional module was identified as the core pivot.

## 3. Results

### 3.1. To Determine the Effect of Preoperative Anesthesia on Gene Expression during an OPCABG

We performed a differential analysis of the gene expression profiles of OPCABG under two kinds of anesthesia to further understand the effect of anesthesia on OPCABG. The 5701 differential genes in the CABG state of sevoflurane anesthesia were obtained, and 3210 differential genes were anesthetized by propofol in the CABG state. We combined the two sets of differences to obtain 6699 differential genes, which were considered to be critical genes for the effects of anesthesia on OPCABG.

### 3.2. Identify Functional Module Networks

**Figure 1:**
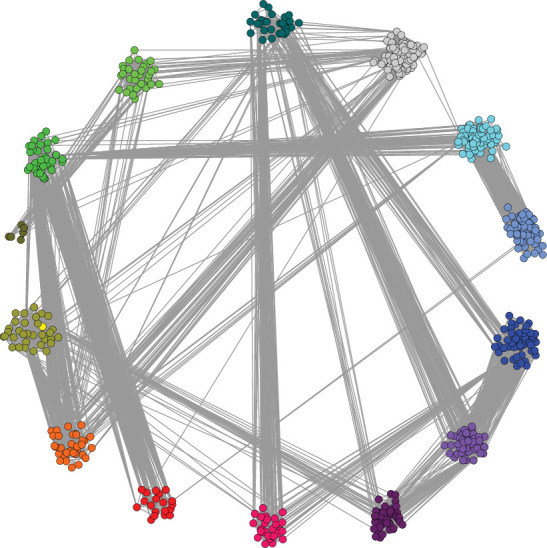
Based on the human interaction protein network, we obtained a protein interaction network map of 6699 differential genes clustered into 14 functional module networks. This network may represent the mechanism of anesthesia affecting nonexternal coronary artery bypass grafting.

Further, we explained the effects of two anesthesia methods on OPCABG from the gene network level. Based on PPI analysis, a protein interaction network was constructed using 6699 differential genes from OPCABG. As a result, a total of 14 interactive networks were obtained, called functional modules (Figure 1). According to functional modules, we identify the critical genes for each module. Analysis of its essential genes may be involved in different functions and pathways, causing different effects of OPCABG in both anesthesia situations.

### 3.3. Functions and Pathways Involved in Specific Genes

**Figure 2:**
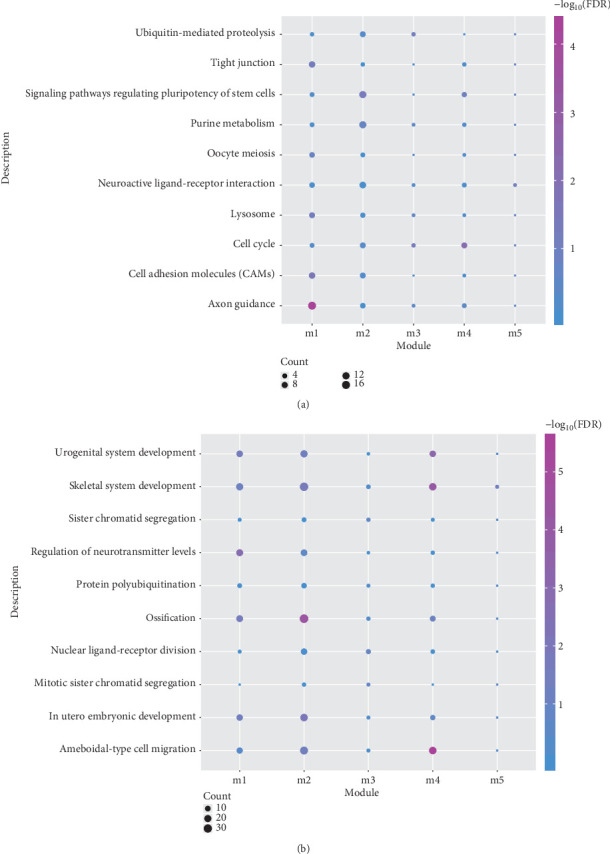
Functional pathways included in modular gene identification of dysfunction modules for osteoarthritis. (a) GO analysis for module genes; (b) KEGG pathway enrichment analysis. The color depth represents the degree of enrichment, and the size of the circle represents the proportion of the genes in the module. FDR: false discovery rate.

To further understand the biological aspects of the module genes, GO function and KEGG analyses were performed on 14 functional module networks. We obtained GO terminology, including 1314 cell composition entries, 1640 molecular functional terms, and 13,160 biological processes (Figure 2(a)). Most of the modular genes are associated with the regulation of the cytokine-mediated signaling pathway and positive regulation of lymphocyte differentiation. The results of KEGG pathway enrichment reveal that the functional module genes are mainly responsible for platelet activation, cytokine-cytokine receptor interaction, and apoptosis-multiple species (Figure 2(b)). The above results of modular gene enrichment are firmly related to OPCABG; we therefore identified the 14 dysfunction modules.

### 3.4. Regulation of TF and ncRNA of Genes Associated with OPCABG under Anesthesia

**Figure 3:**
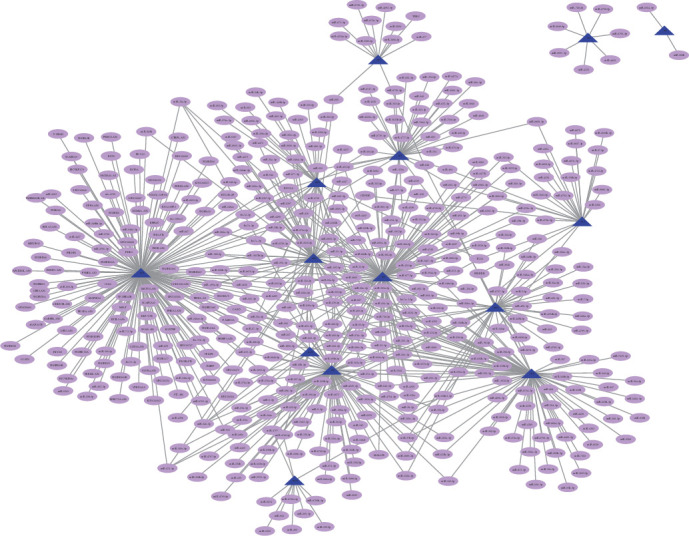
Noncoding RNA (ncRNA) regulatory network of nonexternal coronary artery bypass grafting. The blue triangles represent the module while the pink ovals represent the ncRNA corresponding with the module.

**Figure 4:**
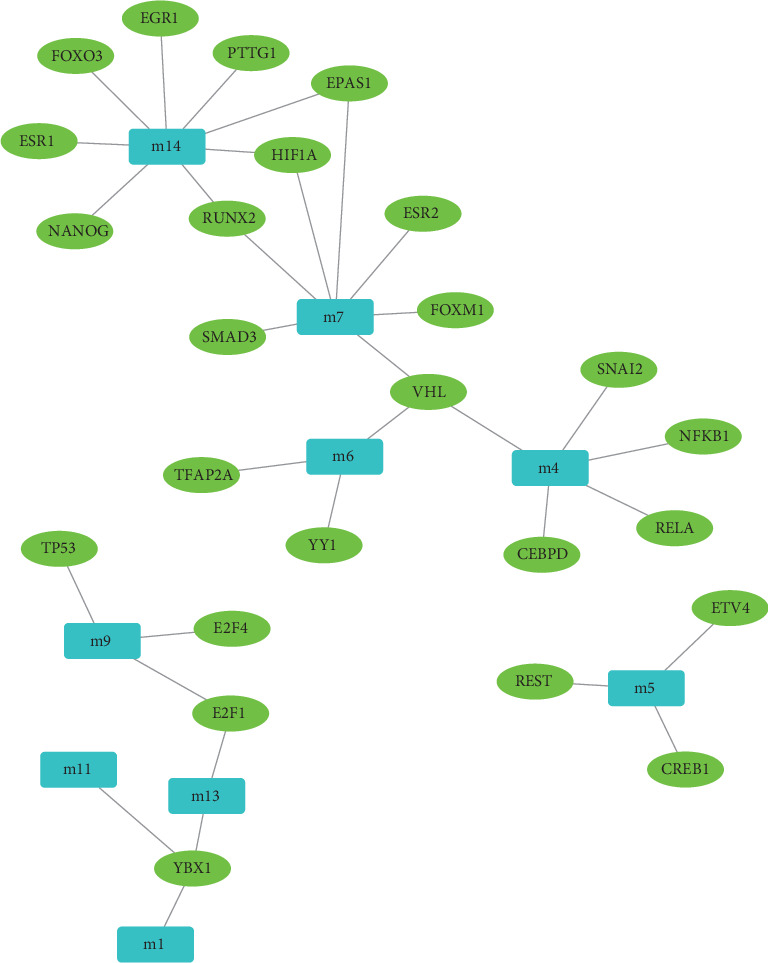
Regulatory network map constructed by transcription factors (TFs) for nonexternal coronary artery bypass grafting. The cyan rectangles represent the module while the green ovals represent the TF corresponding with the module.

To explore the regulatory factors, we applied regulator-predictive analysis to functional modules according to the regulatory relationships in transcriptional and posttranscriptional processes. Based on the number of regulatory modules and the significance of the *p* values, we obtained 486 ncRNAs involving 660 ncRNA-module target pairs and 25 TFs involving 33 TF-module target pairs. Also, we analyzed the predicted results and found that miR-129-5p targets up to six dysfunction modules with a significant impact on OPCABG (Figure 3). The miR-129-5p and miR-410-3p also regulate five dysfunction modules, having remarkable regulatory effects on the module network. VHL and YBX1 have significant regulatory effects on three dysfunction modules and play a key role in the impact of anesthesia on OPCABG (Figure 4). Other TFs also have their specific regulatory effects on the functional module genes, which may have a significant impact on surgery.

## 4. Discussion

In the study of OPCABG, anesthesia can help to reduce intraoperative blood loss and intraoperative blood transfusion demands to varying degrees, shortening hospital stay. The most important is to reduce myocardial enzyme leakage, inflammation, and kidney and nerve damage [23]. However, the use of different anesthesia in OPCABG has different effects on postoperative outcomes. For example, the use of volatile anesthetics to maintain anesthesia in OPCABG surgery contributes to adequate anesthesia depth and reduces the need for analgesia [24]. Helium shows less hemodynamic instability in OPCABG than in conventionally used anesthetics, and it reduces the need for vasopressors [25]. For patients receiving OPCABG, it is important for them to reduce myocardial damage during surgery. The volatility induction and maintenance of sevoflurane has a certain degree of improvement in cardiac function in patients receiving OPCABG. Therefore, compared with intravenous propofol, sevoflurane has a more protective effect on heart damage [26]. The volatile drugs sevoflurane and desflurane can be considered to have a more protective effect on the myocardium of the whole vein [27]. These studies have validated the important role of inhaled anesthesia and intravenous anesthesia in the process of OPCABG, which prompted us to explore the effects of anesthesia in OPCABG. We constructed a PPI network according to differentially expressed RNA data sets for OPCABG after anesthesia and explored 14 functional modules. We found that these modules are mainly involved in positive regulation of lymphocyte differentiation and cytokine-cytokine receptor interaction. The Holler E. study found that after OPCABG, circulating endothelial cell number and apoptotic endothelial cell death are markers of endothelial cell activation and injury. Studies have shown that in OPCABG surgery, in order to protect circulating lymphocytes, the combination of intravenous propofol and gas anesthesia with sevoflurane is superior to the use of sevoflurane to maintain anesthesia [28]. One of the potential advantages of OPCABG is to attenuate systemic inflammatory responses caused by neutrophil activation [29]. Moreover, OPCABG is beneficial to the secretion of endogenous erythropoietin in patients, reducing the blood loss of surgery [30]. Studies have found that OPCABG is associated with decreased troponin I levels and activation, while elevated levels of troponin I and proinflammatory cytokines are present in most cardiac surgery patients [31]. To elucidate the transcriptional regulatory factors of OPCABG by anesthetic gases and intravenous anesthesia, we performed an analysis according to the regulatory relationships both in transcription and posttranscription. It was found that microRNAs (miR-129-5p, miR-340-5p, and miR-410-3p) and transcription factors (VHL and YBX1) have remarkable regulatory effects on functional modules. MicroRNAs play a key role in a variety of cellular processes [32]. miR-129-5p acts on neurons, which have an inhibitory effect on blocking the synaptic reduction in vitro and reducing the severity of seizures in vivo [33]. This also indicates that anesthesia has an important influence on postoperative cell regulation. Abnormal expression of long noncoding RNA plays a crucial part in regulating the progression and drug resistance of human tumors [34]. LGR5 expression was downregulated by the Wnt/*β*-catenin pathway. It leads to overexpression of miR-340-5p, which inhibits apoptosis, cell proliferation, and its resistance [35]. This validated the regulatory changes in genes affected by gene expression after OPCABG. Also, the transcription factor VHL plays a key role in the inactivation of the hypoxia-inducible factor (HIF-1) through regulating the PI3K/AKT/mTOR pathway activity [36]. YBX1 is a multifunctional protein [37]. It is involved in all kinds of DNA/RNA-dependent events, such as DNA repair, mRNA packaging, mRNA transcription and splicing, translational regulation, and mRNA stability. At the cellular level, various activities of YBX1 appear to be involved in stress response, cell differentiation and proliferation, and malignant cell transformation [38]. By regulating cell differentiation, cytokine synthesis, and chemokine synthesis, monocyte YBX1 has a prominent and unique role in cell feed-forward crosstalk and regression of inflammatory processes [39]. Based on the understanding of the anesthetic gas and intravenous anesthesia for the pivot regulator of OPCABG, we can identify the effects of these significant regulators on postoperative outcomes. Different functions are performed in the development of OPCABG after anesthesia.

## 5. Conclusion

A comprehensive functional module network was obtained. The modules, which offer some proven genes and transcription factors to be tested for OPCABG after anesthesia, provides a theoretical basis and reference for further research.

## Data Availability

The data associated with our study was downloaded from GSE4386.
